# The SARS Coronavirus 3a Protein Causes Endoplasmic Reticulum Stress and Induces Ligand-Independent Downregulation of the Type 1 Interferon Receptor

**DOI:** 10.1371/journal.pone.0008342

**Published:** 2009-12-17

**Authors:** Rinki Minakshi, Kartika Padhan, Manjusha Rani, Nabab Khan, Faizan Ahmad, Shahid Jameel

**Affiliations:** 1 Virology Group, International Centre for Genetic Engineering and Biotechnology, New Delhi, India; 2 Centre for Interdisciplinary Research in Basic Sciences, Jamia Millia Islamia, New Delhi, India; University of Hyderabad, India

## Abstract

The Severe Acute Respiratory Syndrome Coronavirus (SARS-CoV) is reported to cause apoptosis of infected cells and several of its proteins including the 3a accessory protein, are pro-apoptotic. Since the 3a protein localizes to the endoplasmic reticulum (ER)-Golgi compartment, its role in causing ER stress was investigated in transiently transfected cells. Cells expressing the 3a proteins showed ER stress based on activation of genes for the ER chaperones GRP78 and GRP94. Since ER stress can cause differential modulation of the unfolded protein response (UPR), which includes the inositol-requiring enzyme 1 (IRE-1), activating transcription factor 6 (ATF6) and PKR-like ER kinase (PERK) pathways, these were individually tested in 3a-expressing cells. Only the PERK pathway was found to be activated in 3a-expressing cells based on (1) increased phosphorylation of eukaryotic initiation factor 2 alpha (eIF2α) and inhibitory effects of a dominant-negative form of eIF2α on GRP78 promoter activity, (2) increased translation of activating transcription factor 4 (ATF4) mRNA, and (3) ATF4-dependent activation of the C/EBP homologous protein (CHOP) gene promoter. Activation of PERK affects innate immunity by suppression of type 1 interferon (IFN) signaling. The 3a protein was found to induce serine phosphorylation within the IFN alpha-receptor subunit 1 (IFNAR1) degradation motif and to increase IFNAR1 ubiquitination. Confocal microscopic analysis showed increased translocation of IFNAR1 into the lysosomal compartment and flow cytometry showed reduced levels of IFNAR1 in 3a-expressing cells. These results provide further mechanistic details of the pro-apoptotic effects of the SARS-CoV 3a protein, and suggest a potential role for it in attenuating interferon responses and innate immunity.

## Introduction

A new virus, the Severe Acute Respiratory Syndrome Coronavirus (SARS-CoV), was responsible for an outbreak of acute respiratory illness in 2003, which affected about 30 countries with over 8000 cumulative infections and more than 900 deaths [1]. The SARS-CoV is a positive-stranded RNA virus with an ∼30 kb genome [2], [3]. Compared to other human and animal coronaviruses, the SARS-CoV genome consists of 9 unique open reading frames (orfs) [4]. Of these, *orf3a* is the largest and encodes a protein of 274 amino acids. The 3a protein is part of the virus particle, is expressed abundantly in infected as well as transfected cells, localizes to intracellular and plasma membranes [5], and induces apoptosis in transfected and infected cells [6], [7].

The endoplasmic reticulum (ER) regulates cellular metabolism and protein synthesis in response to perturbations in protein synthesis and folding. Since the ER is the site for translation and processing of proteins destined for secretion or membrane insertion, many viruses, including the SARS-CoV exploit this organelle. During viral replication there is high biosynthetic burden on the cell for producing viral proteins. The accumulation of nascent and unfolded viral secretory and transmembrane proteins in the ER lumen can lead to ER stress and the downstream activation of multiple signaling pathways [8]. To adjust the biosynthetic burden and capacity of the ER for maintaining cellular homeostasis, the Unfolded Protein Response (UPR) is activated. The UPR is a complex pathway that is mediated by three distinct signaling tracks initiated by the sensors inositol-requiring enzyme 1 (IRE-1), activating transcription factor 6 (ATF6) and PKR-like ER kinase (PERK) [9]. These proteins transduce adaptive signals to the cytosol and nucleus, leading to global effects on ER function [10] and recovery from ER stress. But, prolonged ER stress can also trigger apoptosis.

Viruses have developed various strategies to modulate the UPR [11]–[14]. The hepatitis C virus (HCV) causes increased transcription from the glucose regulated protein 78 (GRP78) and GRP94 promoters through the activation of PERK and ATF6 pathways [15], [16], [17], with simultaneous suppression of the IRE1-X box binding protein (XBP1) pathway [18]. The human cytomegalovirus (CMV) affects UPR through activation of the PERK and IRE-1 branches but spares the ATF6 pathway [19], [20]. A cytopathic strain of bovine viral diarrhea virus (BVDV) induces apoptosis through UPR by activating the PERK pathway [21]. The S protein of SARS-CoV modulates UPR by the transcriptional activation of GRP78/94 and upregulation of the PERK pathway, but has little or no effect on the other two arms of UPR [4]. Since the 3a protein of SARS-CoV is also a transmembrane protein that localizes to the ER-Golgi region and plasma membranes of cells and induces apoptosis, we studied its effects on ER stress and UPR.

Type1 interferon (IFN) signaling exerts anti-proliferative and anti-viral effects through a cell surface cognate receptor consisting of two subunits, the interferon alpha receptor subunit 1 (IFNAR1) and IFNAR2 [22]. Dimerization of these receptor subunits in response to the binding of type I interferons (IFNα or IFNβ) causes the activation of Janus kinase (Jak) family members, Jak1 and Tyk2, which eventually activate the signal transducers and activators of transcription (Stat1 and Stat2) proteins [23]. The Stat proteins cause transcriptional activation of IFN-regulated genes. The cell surface downmodulation and ensuing degradation of the IFN receptor in response to IFNα treatment is a crucial event in limiting the extent of interferon-mediated cellular responses [24], [25]. The mechanism involves ligand-induced Tyk2 activity-dependent [26], [27], [28], [29] or ligand-and Jak-independent [30] phosphorylation of the Ser535 residue in the degradation motif (degron) of IFNAR1, which is followed by its ubiquitination and lysosomal degradation [26].

Activation of the PERK arm of UPR affects innate immunity of a cell by accelerating degradation of the IFNAR1 subunit; during infections by vesicular stomatitis virus (VSV) and the hepatitis C virus (HCV) this reduces the sensitivity of the infected cell to IFN [31]. The degradation signal is phosphorylation of a serine residue within the IFNAR1 degron, which leads to increased ubiquitination and receptor degradation through the lysosomal pathway in virus infected cells [31]. In the current study we present evidence that the 3a protein of SARS-CoV elicits UPR by modulating the PERK pathway, and this leads to phosphorylation, ubiquitination and lysosomal degradation of IFNAR1.

## Results

### The SARS-CoV 3a Protein Induces ER Stress

The SARS-CoV utilizes the ER for processing its structural and nonstructural proteins [32]. During viral replication, substantial amounts of viral transmembrane glycoproteins such as the spike (S) and matrix (M) proteins are produced [32], of which the S protein induces ER stress [4]. Since the 3a protein is also a transmembrane glycoprotein that localizes to the ER-Golgi compartments, we studied its potential to induce ER stress.

**Figure 1:**
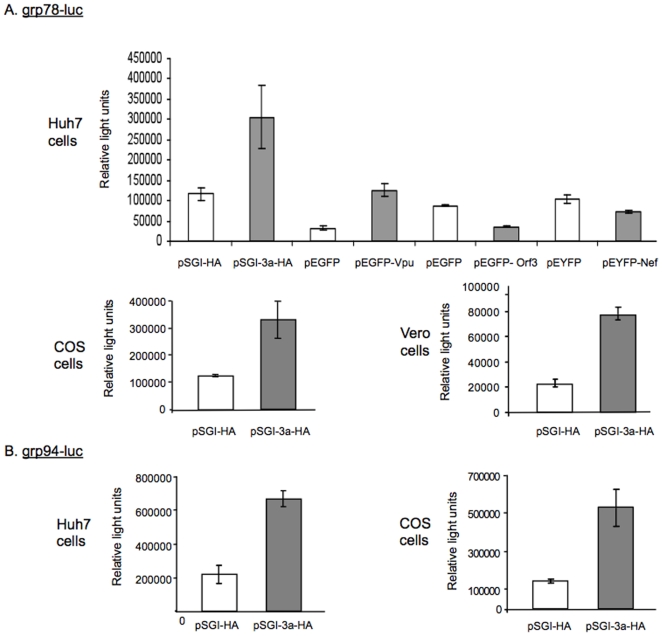
Induction of ER stress by the 3a protein. (A) The grp78-luc reporter plasmid (0.25 µg) was co-transfected in Huh7 with plasmid pSGI, pEGFP or pEYFP (vector controls) or pSGI-3a-HA, pEGFP-Vpu, pEGFP-Orf3 or pEYFP-Nef (0.75 µg each). COS or Vero cells were also co-transfected with grp78-luc (0.25 µg) and either plasmid pSGI-HA (vector control) or pSGI-3a-HA (0.75 µg each). (B) The grp94-luc reporter plasmid (0.25 µg) was similarly co-transfected in Huh7 or COS cells along with plasmid pSGI-HA (vector control) or pSGI-3a-HA (0.75 µg each). The transfections were carried out in 12-well plates. Cell lysates were prepared 48 hr post-transfection, the protein content estimated and the normalized lysates were analyzed for luciferase activity, expressed as Relative Light Units. Each bar is representative of three separate experiments, each with three independent transfections.

To investigate this, we looked at the transcriptional activation of grp78 and grp94 genes, which encode crucial ER chaperones that are markers of ER stress, in cells expressing the 3a protein. To rule out induction of ER stress merely due to over-expression of a viral protein, we also studied the effects of the HIV proteins Nef and Vpu, and the hepatitis E virus (HEV) Orf3 protein, on grp78 and grp94 activation. A luciferase reporter construct driven by the grp78 promoter and either plasmid pSGI, pEGFP, pEYFP (vector controls) or pSGI-3a-HA, pEGFP-Vpu, pEGFP-Orf3 or pEYFP-Nef were transiently transfected into Huh7 cells. The cells were harvested 48 hr post-transfection for measurement of luciferase activity. We observed about 3 to 4 fold increase in luciferase activity in 3a expressing cells as compared to the vector control cells. The transient expression of HIV Vpu also gave a similar increase in grp78 promoter activity, HIV Nef did not show any effect, and HEV Orf 3 showed about 2.5 fold reduction in grp78 promoter activity (Fig. 1A). We also tested the effects of the SARS-CoV 3a protein on grp78 promoter in two other cell lines, COS and Vero cells, and observed similar effects as in Huh7 cells (Fig. 1A). Activation of the grp94 promoter by the SARS-CoV 3a protein was also tested in Huh7 and COS cells. This promoter was also activated about 3 to 4 folds by expression of the 3a protein (Fig. 1B). These results using two different ER sensor reporters, multiple cell lines and other viral proteins as controls, suggest that the 3a protein causes ER stress.

### The 3a Protein Differentially Activates the PERK but Not the IRE-1 and ATF6 Branches of UPR

**Figure 2:**
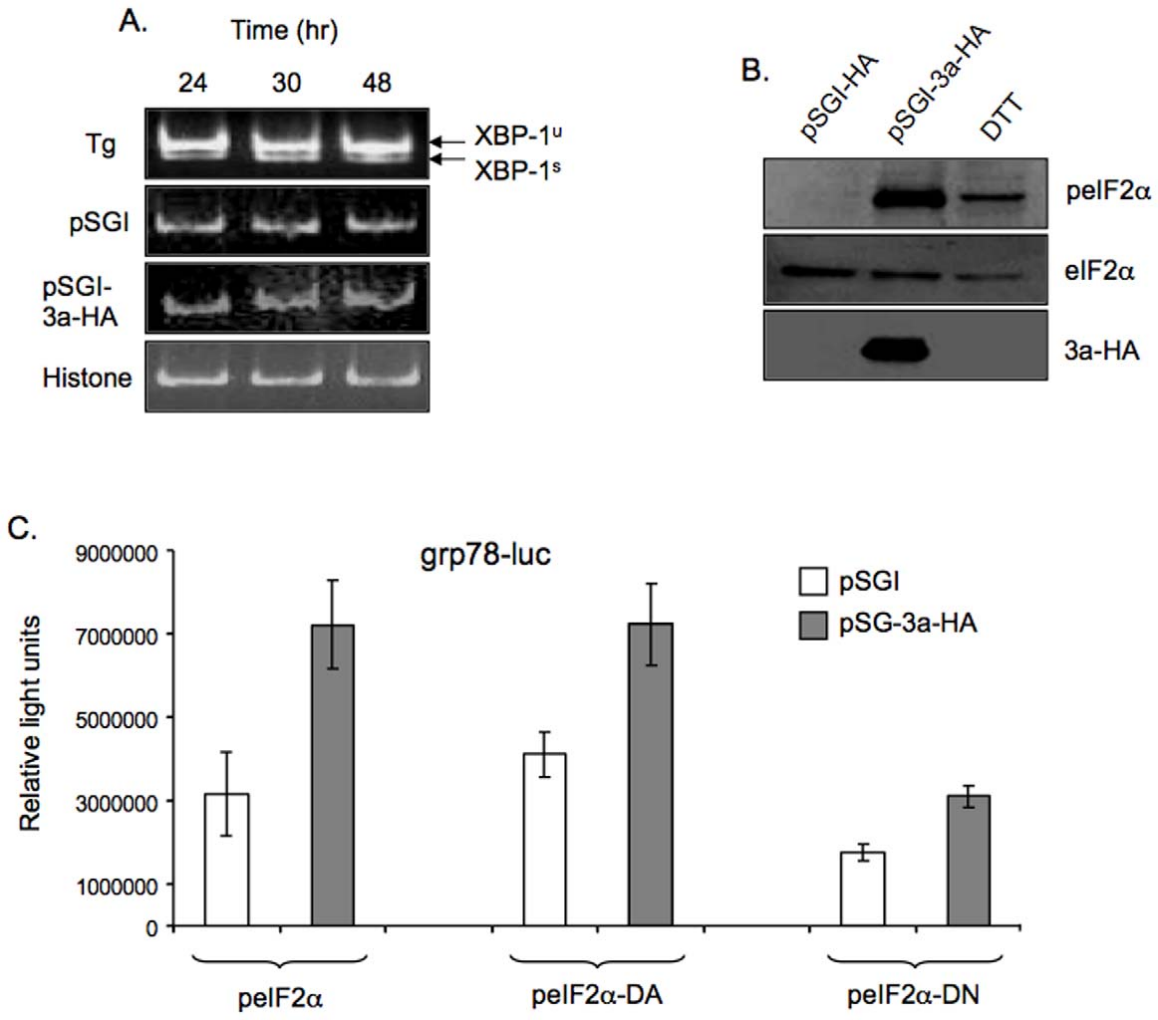
The 3a protein mediates ER stress through eIF2α. (A) Huh7 cells were transfected with 1 µg of either pSGI or pSGI-3a-HA, or untransfected cells were treated with 500 nM thapsigargin (Tg) for 16 hr prior to harvest. The cells were harvested at the indicated times, RNA prepared with Trizol and amplified by RT-PCR for the XBP-1 unspliced (XBP-1^u^) and spliced (XBP-1^s^) RNA forms. RT-PCR for histone mRNA was used as a loading control. (B) Huh7 cells were transfected with 1 µg of either pSGI or pSGI-3a-HA; untransfected cells were treated with 1.5 mM dithiothreitol for 45 min prior to harvest, as a positive control for ER stress. Cell lysates were prepared 48 hr post-transfection, equal amounts of proteins were separated by SDS-PAGE and western blotted for the indicated proteins. (C) Huh7 cells were co-transfected with the grp78-luc reporter plasmid (0.2 µg), pSGI or pSGI-3a-HA (0.4 µg), and an expression plasmid for either wild type, dominant-active (DA) or dominant-negative (DN) eIF2α (0.4 µg). After 48 hr, cell lysates were prepared, normalized and analyzed for luciferase activity. Each bar is representative of two separate experiments, each with three independent transfections. All the transfections were carried out in 12-well plates.

Since ER stress leads to activation of UPR, which comprises of three different branches, we tested for the effects of 3a expression on each of these. The IRE-1 and ATF6 pathways lead to reprogramming of the cell and induction of ER associated degradation [33], [34], [35]. Following the activation of IRE-1 by ER stress, its endoribonuclease activity causes unconventional splicing of the mRNA for the X box-binding protein 1 (XBP-1). To determine the effect of 3a expression on the IRE-1 pathway, we carried out reverse transcription-polymerase chain reaction (RT-PCR) for the spliced XBP-1 mRNA as described elsewhere [36]. The spliced XBP-1 RNA was not observed in vector control or 3a-expressing cells, but was present in cells treated with thapsigargin, a chemical inducer of ER stress (Fig. 2A). During ER stress, ATF6 is translocated to the Golgi where its cytoplasmic domain (ATF6f) is liberated by regulated proteolysis [37], [38]. To study the effect of 3a expression on the ATF6 pathway, we looked at the levels of ATF6f following cotransfection of a FLAG-tagged ATF6 expression plasmid that mimicked ATF6 processing upon induction of ER stress, and either the empty pSGI vector or the pSGI-3a-HA expression vector. We did not observe any significant increase in the cleaved ATF6f (p50) product arising from transfected FLAG-tagged ATF6 (p90) in 3a expressing cells as compared to control cells; however, treatment with thapsigargin led to increased levels of the p50 form (not shown).

Under conditions of ER stress the kinase activity of PERK is activated, phosphorylating its downstream target, the eukaryotic translation initiation factor 2 subunit α (eIF2α). To test if 3a protein-mediated ER stress was through the PERK pathway, we first looked at the phosphorylation status of eIF2α by western blotting of transfected cell lysates. Compared to control cells, the 3a-expressing cells showed increased levels of phospho-eIF2α (peIF2α) without a concomitant increase in total eIF2α levels (Fig. 2B). We then cotransfected cells with expression plasmids for wild type eIF2α or its dominant-negative (DN) or dominant active (DA) mutants, pGRP78-Luc and either pSGI or pSG-3a-HA. Since the DN form of eIF2α constitutively inhibits phosphorylation of eIF2α, the promoter activity of GRP78/94 would be compromised. We observed that the induction of ER stress in 3a expressing cells, measured with the grp78-luc reporter, was compromised in the presence of eIF2α DN (Fig. 2C), confirming the role of 3a protein in activating the PERK-eIF2α pathway.

**Figure 3:**
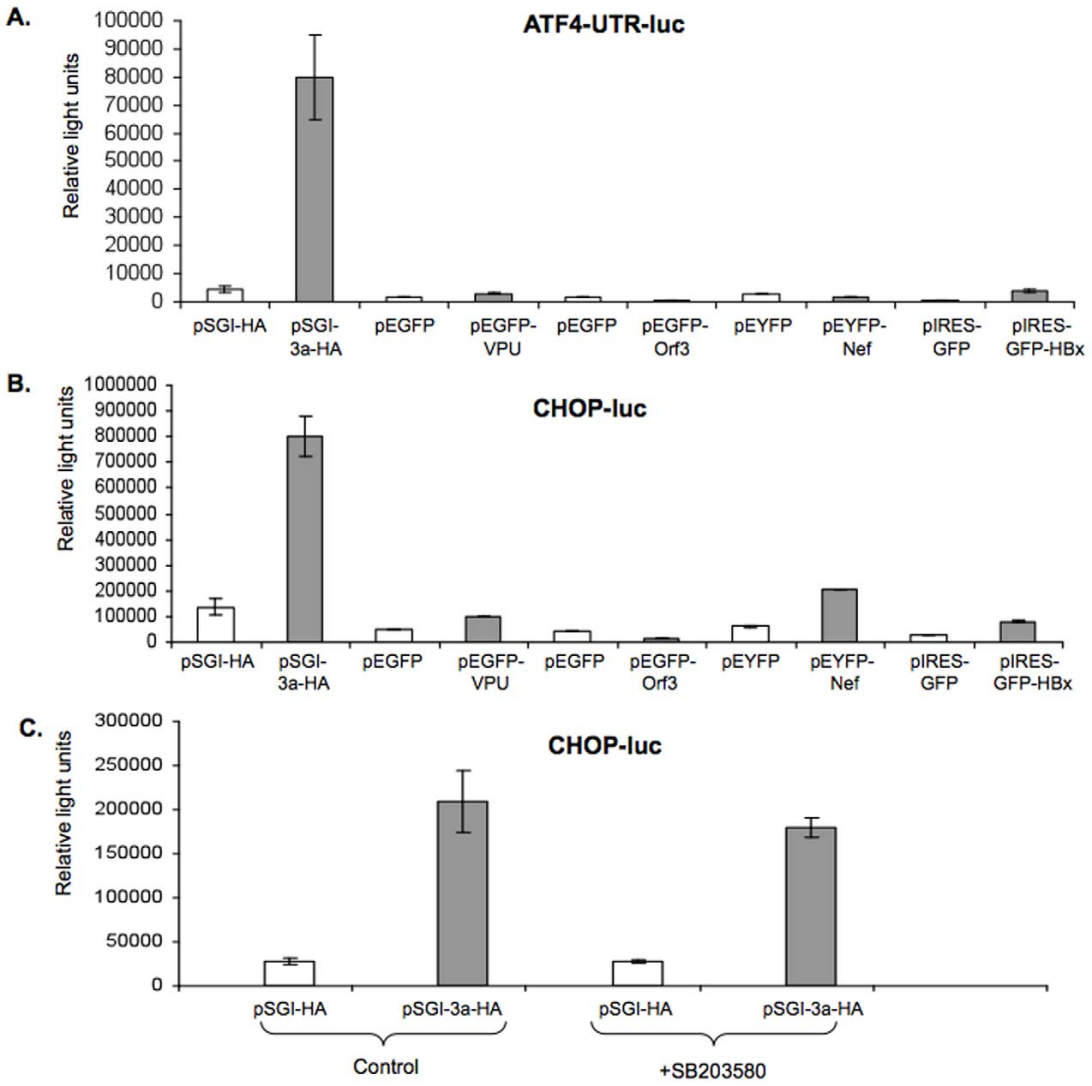
The 3a protein activates the PERK pathway. The (A) ATF4-UTR-luc or (B) CHOP-luc plasmids (0.25 µg each), which express luciferase under control of the ATF4 untranslated region or the CHOP gene promoter, respectively, were co-transfected in Huh7 cells together with 0.75 µg of plasmid pSGI-HA, pEGFP, pEYFP or pIRES-EGFP (as controls) or pSGI-3a-HA, pEGFP-Vpu, pEGFP-Orf3, pEYFP-Nef, pIRES-GFP-XO (as test). (C) The grp78-luc reporter plasmids (0.25 µg each) were co-transfected in Huh7 with either plasmid pSGI-HA (vector control) or pSGI-3a-HA (0.75 µg each), except that one set of cells were treated with 20 µM SB203580 for 12 hr before harvest, while the controls were treated with the same volume of DMSO. Cell lysates were prepared 48 hr post-transfection, and normalized lysates were analyzed for luciferase activity. Each bar is representative of two separate experiments, each with three independent transfections, which were carried out in 12-well plates. In (C), the p value for comparisons between untreated and SB203580-treated pSGI-3a-HA transfected cells was calculated to be 0.345.

While PERK-mediated phosphorylation of eIF2α leads to a decrease in its activity, it also causes a paradoxical increase in translation of ATF4 mRNA [39], [40], [41]. The ATF4 protein is a transcription factor that binds to and activates the promoter of C/EBP homologous protein (CHOP, also called as GADD153) gene [39], [40], [41]. To investigate the effect of 3a and other viral proteins on downstream effectors of the PERK pathway, we employed the construct TK.mATF4.UTR.Luc.pGL3, which contains the 5′-untranslated region of ATF4 fused to the coding region of firefly luciferase, for studying ATF4 translation in transiently transfected cells. There was about 17-fold increase in ATF4 expression in 3a-expressing cells compared to control cells; the other viral proteins did not show any significant effect on ATF4 translation (Fig. 3A). The activity of the CHOP promoter was also assayed in cells cotransfected with the pSGI-3a-HA expression construct (or pSGI) and a luciferase reporter construct driven by CHOP promoter. There was an ∼4-fold increase in CHOP promoter activity in 3a-expressing cells (Fig. 3B); the other viral proteins showed variable results. The HEV Orf3 protein showed a decrease in CHOP promoter activity, whereas the HIV Nef and Vpu proteins and the HBV X protein showed marginal increases in the CHOP promoter activity (Fig. 3B).

Since activation of the CHOP promoter can also be mediated by the p38 MAPK pathway and our earlier results show that the 3a protein can also activate this pathway, we employed SB203580, a chemical inhibitor of the p38 MAPK to dissect the effects of the 3a protein on CHOP activation. The CHOP promoter-reporter construct was cotransfected into cells with either the 3a expression plasmid (or the vector control plasmid). One set of transfected cultures was treated with SB203580, while the other was treated with the vehicle (DMSO). Luciferase assays carried out on cell lysates did not reveal any significant effect of SB203580 on the activity of the CHOP promoter in 3a expressing cells at concentrations that inhibit p38 activity (Fig. 3C). This shows that transcriptional activation of CHOP expression by the 3a protein was independent of its activation of the p38 MAPK pathway.

### The 3a Protein Promotes Phosphorylation, Ubiquitination and Lysosomal Degradation of the Interferon-Alpha Receptor Subunit 1 (IFNAR1)

**Figure 4:**
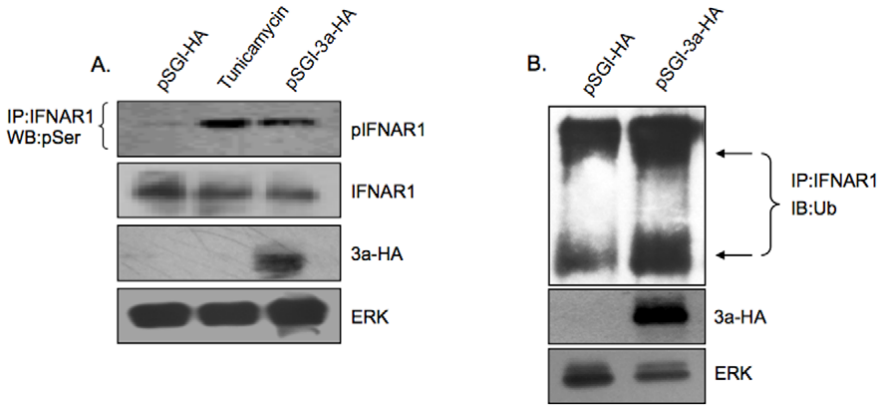
The 3a protein increases phosphorylation of IFNAR1 and its subsequent ubiquitination. (**A**) Huh7 cells were transfected with plasmid pSGI-HA or pSGI-3a-HA (2 µg each); untransfected cells were also treated with 1 µM tunicamycin for 16 hr before harvest as a positive control for ER stress. Cell lysates were prepared 48 hr post-transfection and equal amounts of proteins were subjected to immunoprecipitation (IP) using IFNAR1 antibody followed by western blotting with a phosphoserine antibody. Cell lysates were also subjected to western blotting with indicated antibodies as controls. (B) Transfections were as in (A) above. Normalized cell lysates were subjected to immunoprecipitation (IP) with an anti-IFNAR1 antibody followed by western blotting (WB) with an anti-Ubiquitin antibody. Cell lysates were also directly subjected to western blotting with indicated antibodies as controls. Arrows indicate the partially (lower band) and heavily glycosylated (higher band) forms of the IFNAR1 protein. The transfections were carried out in 60 mm dishes.

The activation of PERK causes phosphorylation of IFNAR1 within its degron region; this is important for its ubiquitination and downregulation at the cell surface [31]. To investigate the fate of IFNAR1 in 3a expressing cells, we first looked at its phosphorylation levels. Increased IFNAR1 phosphorylation was observed in 3a expressing cells, compared to control cells (Fig. 4A). As a positive control, tunicamycin, a chemical inducer of ER stress also showed increased IFNAR1 phosphorylation. There was also a slight decrease in the steady state levels of the IFNAR1 protein in cells that were either treated with tunicamycin or expressed the 3a protein (Fig. 4A). This would indicate either decreased synthesis or increased degradation of the IFNAR1 protein under these conditions. Since IFNAR1 phosphorylation is established as a signal for its ubiquitination, we tested for that by immunoprecipitating lysates from transiently transfected cells with anti-IFNAR1 antibodies and then western blotting the precipitates with an anti-ubiquitin antibody. Compared to control cells, the 3a expressing cells showed increased ubiquitination of the IFNAR1 protein (Fig. 4B).

**Figure 5:**
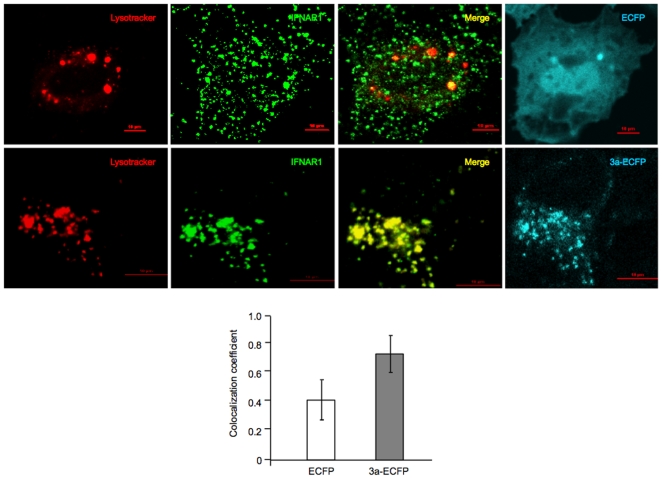
The 3a protein increases lysosomal accumulation of IFNAR1. Huh7 cells were grown on coverslips placed in the wells of 6-well dishes and transfected with 2.5 µg of plasmid pECFP or p3a-ECFP. After 48 hr, the cells were washed and stained with Lysotracker as well as an anti-IFNAR1 antibody, as described in Materials and Methods. The cells were then imaged with a confocal microscope, the images quantitated and colocalization coefficients calculated as described. Separate images for Lysotracker (red), IFNAR1 (green) and ECFP/3a-ECFP (cyan) are shown, together with merged images for Lysotracker and IFNAR1. Scale bar, 10 µm. The bar graph shows Pearson's colocalization coefficients; p = 1.8×10^−7^.

Ubiquitinated IFNAR1 is destined to undergo degradation through a lysosomal pathway [26], [42], [43]. To test for this, we took cells transiently expressing either the enhanced cyan fluorescent protein (ECFP) as a control or the 3a-ECFP-fusion protein, and co-stained these cells with an antibody to IFNAR1 and Lysotracker. Multiple cells were imaged by confocal microscopy and colocalization of the IFNAR1 and Lysotracker signals was quantitated. There was increased localization of IFNAR1 in the lysosomal compartment in 3a expressing cells compared to control cells (Fig. 5).

**Figure 6:**
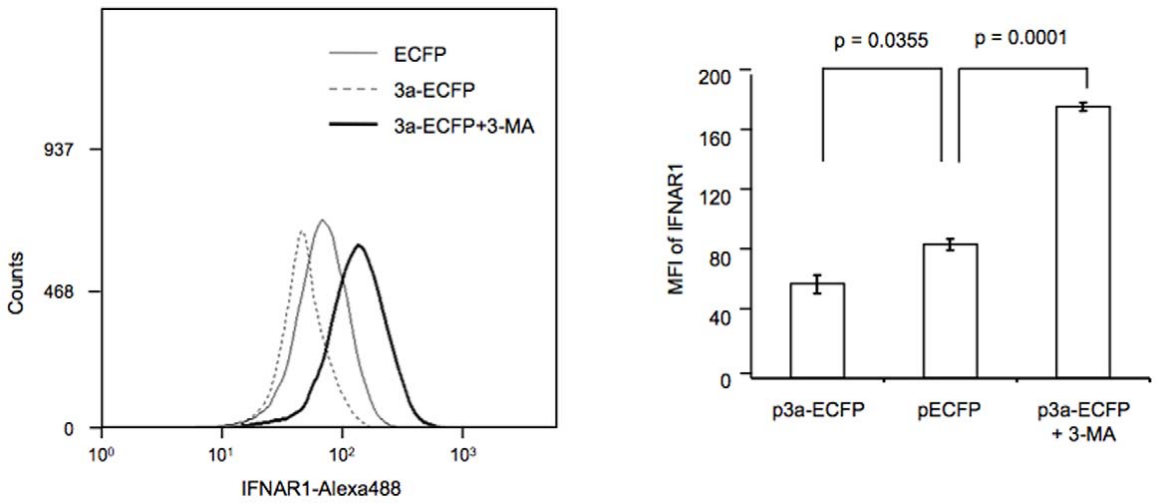
The 3a protein reduces endogenous levels of IFNAR1. Huh7 cells in 60 mm dishes were transfected with 2.5 µg of either pECFP or p3a-ECFP. In one set, the p3a-ECFP transfected cells were also treated with 20 mM 3-MA for 1 hr prior to harvest. The cells were harvested 48 hr post-transfection and endogenous levels of IFNAR1 were analyzed by flow cytometry as described in Materials and Methods. The mean fluorescence intensity (MFI) of IFNAR1 signal was estimated and p values shown were calculated with the Student's T test. The flow cytometry plot is representative of three separate experiments, while the MFI values shown are an average (±SD) of these experiments.

Increased ubiquitination and lysosomal translocation of IFNAR1 in 3a expressing cells is likely to result in reduced IFNAR1 levels in these cells. We tested this by staining for IFNAR1 in a flow cytometric assay using cells transiently expressing either the control ECFP or the 3a-ECFP-fusion protein. As shown in Fig. 6, there was a significant decrease in the equilibrium levels of IFNAR1 in 3a-ECFP expressing cells compared to control cells. To verify involvement of the lysosomal pathway in reduced IFNAR1 levels, we also treated 3a-ECFP expressing cells with the lysosomal inhibitor, 3-methyl adenine (3-MA), which led to an increase in the cellular IFNAR1 signal (Fig. 6), thus confirming a role for the lysosomal pathway in its reduction in 3a expressing cells.

## Discussion

The 3a protein of SARS-CoV has multiple functions. It has an ion channel activity [44], modulates trafficking properties of SARS-CoV spike (S) protein [5], upregulates the expression of fibrinogen in A549 lung epithelial cells [45] and induces apoptosis in various cell types *in vitro* and *in vivo* [6], [46].

All viruses encode proteins that are synthesized by the host cell and are processed through the cellular protein translocation machinery. This puts an added burden on the ER, resulting in ER stress. To overcome the effects of ER stress, many viruses, including coronaviruses, modulate the UPR [47]. The S protein of SARS-CoV that forms its surface coat, is produced in large amounts in infected cells and is trafficked through the ER, modulates UPR [4]. The 3a protein is another viral transmembrane glycoprotein expressed in SARS-CoV infected cells, which has previously been shown to localize to the ER-Golgi network and plasma membrane in cells [5], and to viral particles. We show here that the 3a protein also induces UPR.

In the cell, UPR progresses through three different pathways that are mediated by IRE-1, ATF6 and PERK. The IRE-1 and ATF6 pathways lead to reprogramming of the cell and induction of ER associated degradation, which reduces burden on the ER [33], [34], [35]. IRE-1 is a type I transmembrane protein kinase endoribonuclease [48], [49] containing three ER stress-regulated domains, which include a lumenal dimerization domain, a cytosolic kinase and an endoribonuclease [50]. Following ER stress, IRE-1 dimerizes and undergoes autophosphorylation and activation of its endoribonuclease activity. This causes unconventional splicing of the IRE-1 effector X box-binding protein 1 (XBP-1) mRNA that encodes XBP-1, a basic leucine zipper (bZIP) transcription factor and belongs to ATF/CREB family [51], [52], [53]. The ATF6 is a type II transmembrane protein whose cytosolic domain is a bZIP transcription factor [37]. During ER stress it is translocated to the Golgi [54], where its cytoplasmic domain (ATF6f) is liberated by regulated proteolysis and translocates into the nucleus to activate transcription [37], [38]. Our results show neither XBP-1 mRNA splicing nor ATF6 proteolysis in 3a expressing cells, suggesting that the IRE-1 and ATF6 pathways are not activated in these cells.

We did however find activation of the PERK pathway of UPR by the 3a protein. On activation, the PERK kinase causes phosphorylation of eIF2α, which leads to reduced translation of cellular proteins, but stimulates expression of ER molecular chaperones such as GRP78 and GRP94 [55]. Modulation of UPR by the 3a protein represents another function aimed at regulating the cellular response. Increased transcriptional activation and biosynthesis of ER chaperones would enhance folding of the 3a and other viral proteins in the ER lumen. The induction of Endoplasmic Reticulum Associated Degredation (ERAD) to reduce the protein-folding load on the ER requires activation of the IRE1/XBP1 and ATF6 pathways. This includes ER degradation-enhancing α-mannosidase-like protein (EDEM), which is directly involved in recognition and targeting of unfolded proteins for degradation during UPR [33]. By activating only the PERK, but not the IRE-1 and ATF6 pathways during UPR, the 3a protein potentially protects itself and other viral proteins from ERAD, while promoting the expression of ER chaperones and folding of proteins that have accumulated in the ER.

We showed earlier that the 3a protein activates p38 MAPK and is proapoptotic [7]. The expression of C/EBP homologous protein (CHOP), which is known to have a pro-apoptotic effect, is activated by p38 MAPK as well as through the PERK signaling pathway [56], [57]. The over-expression of CHOP leads to translocation of Bax from the cytosol to the mitochondria [58]. The CHOP-mediated death signal is finally transmitted to the mitochondria, which functions as an integrator and amplifier of the death pathway but the other mechanistic details of CHOP action are still unclear [57]. We show the 3a protein to cause increased phosphorylation of eIF2α, which is indicative of activation of the PERK arm of UPR [55], [59]. The activation of downstream effectors such as ATF4 and CHOP indicates yet another mechanism through which the 3a protein can be pro-apoptotic. Since the p38 MAPK inhibitor SB203580 showed no significant effect on transcriptional activation of the CHOP promoter in 3a expressing cells, the p38 MAPK pathway is not likely to mediate these effects of the 3a protein. Overall, our results show that 3a-expressing cells respond to ER stress by activation of the PERK pathway, which enhances protein folding in the ER instead of activating ERAD, the latter being detrimental to virion biosynthesis. However, prolonged PERK activation would lead to apoptosis.

Since our approach was based on transient transfection, it was important to rule out ER stress effects simply due to over-expression of the SARS-CoV 3a protein. Therefore other viral proteins were also tested for upstream (grp78 promoter activation) and downstream (ATF4, CHOP) markers of ER stress. The HIV-1 Vpu protein being a type I transmembrane protein [59] caused grp78 promoter activation but it did not significantly affect downstream effectors of the PERK pathway. The HIV-1 Nef protein, a membrane-anchored protein also did not show any significant effects on ER stress. The HBV X protein has been shown to cause ER stress [60]; in our studies it caused mild activation of ATF4 and CHOP. Finally, the HEV Orf3 protein, which associates with cytoskeletal and mebrane fractions [61], showed a reduction in ER stress measured through the transcriptional activation of grp78 and to a lesser extent that of CHOP. Interestingly, another protein of this virus, the Orf2 protein, was earlier shown to cause ER stress [62]. Thus, our results clearly show specific activation of ER stress and the PERK signaling pathway by the SARS-CoV 3a protein.

The co-evolution of viruses with their hosts has also led to viral evasion strategies, including those that limit the effects of innate and adaptive immunity [63]. An important innate response following viral infection is the synthesis and action of interferons [64]. Interferons (IFN) are synthesized and secreted by infected cells, and act on neighboring cells to elicit a variety of protective responses that limit the further spread of viral infection [64]. For interferons to act on cells, they must first bind their cognate receptors on the cell surface [65], [66]. Not surprisingly, therefore, one viral evasion mechanism is down regulation of the IFN receptor. Recently, ligand-independent downregulation of type I IFN signaling was observed in virus-infected cells [31]. This involved PERK activation during UPR causing the phosphorylation-dependent ubiquitination and degradation of IFNAR1. Since the 3a protein also activated PERK and its downstream effectors, we tested the effects of this viral protein on IFNAR1. Our results showed ligand (IFN)-independent phosphorylation and ubiquitination of IFNAR1 in 3a expressing cells, increased translocation of IFNAR1 to the lysosomal compartment for degradation and reduced levels in these cells.

The hepatitis C virus (HCV) expresses viral proteins that cause ER stress [67], and HCV-induced UPR promotes IFNAR1 phosphorylation, ubiquitination and downregulation [31]. The vesicular stomatitis virus (VSV) also evokes a similar response [31]. Our results suggest that the SARS-CoV 3a protein might specifically inhibit type I IFN signaling and antiviral defenses thereby making 3a expressing or virus-infected cells less sensitive to IFNα. Interestingly, the treatment of SARS patients with only IFNα is also reported to have shown low efficacy during the 2003 outbreak [68].

We propose here that the 3a protein of SARS-CoV induces degradation of IFNAR1 and elicits apoptotic conditions by transcriptional upregulation of CHOP through differential activation of the PERK arm of UPR. Further studies are needed, especially in an animal model of disease, to confirm the extent of these effects in SARS-CoV pathogenesis.

## Materials and Methods

### Materials, Plasmids and Primers

All common reagents were from Sigma Chemical Co. (St. Louis, MO, USA) unless stated otherwise. COS-1 and Vero cells were obtained from American Type Culture Collection (Manassas, Va, USA), and Huh7 cells were from Dr. J. Sato, Okayama University, Japan. All cell lines were cultured at 37°C in 10% CO_2_ in complete Dulbecco modified Eagle medium (DMEM containing 1 g/lit glucose, 2 mM L-glutamine, 1.5 g/lit sodium bicarbonate, 0.1 mM non-essential amino acids, 0.1 mg/ml streptomycin, 100 U penicillin) and 5% fetal bovine serum (FBS). Antibodies generated against a recombinant 3a protein have been described previously [69]. The ATF6 and IRE-1α reagents were kindly provided by Ron Prywes, Columbia University, New York [70], the ATF4 and eIF2α reagents were from David Ron, NYU Medical Center, New York, the pCHOP-Luc reporter plasmid containing human C/EBP homologous protein (CHOP) promoter was from Nai Sum Wong, Faculty of Medicine, University of Hong Kong [71], and the pGRP78-Luc and pGRP94-Luc reporter constructs were from Kazutoshi Mori, Kyoto University, Japan [72]. Antibodies to phospho-eIF2α and eIF2α were from Cell Signaling Technology and the IFNAR1 and anti-Ubiquitin antibodies were from Santa Cruz Biotechnology. The cloning of orf3a and construction of the pSGI-3a-HA and p3a-ECFP expression vectors has been described previously [69]. The cloning of other viral proteins like Nef [73], Vpu [74], HEV Orf 3 [75] and HBV X [76] has been described elsewhere. The primers for amplifying unspliced (u) and spliced (s) forms of XBP-1 transcripts were: 5′-TTACGAG AGAAAACTCATGGC-3′ and 5′-GGGTCCAAGTTGTCCAGAATGC-3′, and amplification was according to the protocol described elsewhere [77]. Histone transcripts were amplified with the primers 5′-TGAGAGACAACATTCAGGGCATCAC -3′ and 5′-CGCTTGAGCGCGTACACCACATCCAT-3′.

### Transfection and Immunoblotting

Tranfections were done with the Fugene 6 reagent (Roche, Mannheim, Germany) according to the manufacturer's instructions. At the indicated times post-transfection, cells were scraped, lysed and processed as described previously [7]. The protein concentration of the lysate was estimated using Bradford regeant (Bio-Rad), and immunoblotting was carried out as described previously [69].

### Luciferase Reporter Assays

Transfected cells were harvested after 48 hrs and lysed in the cell lysis buffer (Luciferase Assay Kit; Promega, Madison, WI, USA). Normalized cell lysates were analyzed for luciferase activity according to the supplier's protocol and readings were taken on a luminometer (Sirius, Berthold, Germany).

### Immunoprecipitation

Transfected cells were lysed in a buffer containing 20 mM Tris-HCl, pH 7.5, 150 mM NaCl, 1 mM EDTA, 1 mM EGTA, 1% Triton X-100, 1 mM NaF, 1 mM sodium orthovandate and a protease inhibitor cocktail (Roche, Mannheim, Germany). The lysates were clarified by centrifugation at 16,000×g for 20 min at 4°C. Protein quantitation was done by Bradford assay (Bio-Rad Laboratories). For immunoprecipitation of phosphoprotein, 0.7 to 1 mg of total protein in 400–500 µl lysis buffer was first incubated with 20 µl of Protein G-agarose beads (GE Healthcare, Uppsala, Sweden) for 1 hr at 4°C. The pre-cleared lysate was then incubated with 2 µg of the antibody overnight at 4°C, followed by 20 µl of equilibrated Protein G-agarose beads for 3 hr at 4°C. After washing five times in lysis buffer, the beads were boiled in Laemmli buffer, the proteins were separated by SDS-PAGE, followed by immunoblotting as described previously [69].

### Imunofluorescence and Subcellular Localization

For transient transfection, cells were grown on coverslips to 40–50% confluence and transfected with Fugene 6 (Roche, Mannheim, Germany). Around 48 hr post-transfection, the cells were incubated with 100 nM Lysotracker (Molecular Probes, USA) in growth medium for 3 hr in dark, at 37°C in a 10% CO_2_ incubator. The cells were then washed with PBS and fixed with 2% paraformaldehyde for 15 min at room temperature. For antibody staining, cells were permeabilized with 0.4% saponin for 15 min at room temperature, blocked with PBS containing 5% bovine serum albumin (BSA) for 30 min and then incubated with a 1∶50 dilution of the primary antibody in PBS containing 5% BSA for 1 hr at room temperature. The cells were washed thrice for 5 min each with PBS and then incubated with a 1∶500 dilution of the relevant Alexa 647 conjugated secondary antibody (Molecular Probes, USA) in PBS containing 5% BSA for 1 hr. The coverslips were mounted on slides with Antifade reagent (Bio-Rad, Hercules, USA) and sealed with a synthetic rubber-based adhesive (Fevicol; Pidilite Industries, India). Imaging and analysis was done on a Nikon A1R confocal microscope. The Pearson coefficient for colocalization was calculated using the Nikon Elements Software.

### Flow Cytometry

Huh7 cells were transfected as above and 48 hr later the cells were washed twice with PBS. Subsequently, 1.5 to1.8×10^6^ cells were harvested in 1 mM EDTA in PBS for 1 min following by two washings with PBS. The cells were fixed with 2% paraformaldehyde (PFA) for 20 min at 4°C, washed twice with PBS, then resuspended in permeabilization buffer (PB; 0.1% Saponin and 1% fetal calf serum in PBS) for 10 min at 4°C followed by two washings with PB. The cells were then incubated in PB containing 1∶50 diluted rabbit anti-IFNAR1 for 30 min at 4°C. After washing twice with PB, the cells were incubated with 1∶100 diluted Alexa-488 conjugated anti-rabbit IgG (Molecular Probes, USA) for 30 min in dark at 4°C. Lastly, the cells were washed thrice with PB and kept in staining buffer (0.1% PFA, 1% FCS in PBS). For each sample, 25,000 events were acquired using a CyAn-ADP flow cytometer (Dako) and the data analyzed with Summit software (version 4.3). The cells were gated for ECFP or 3a-ECFP expression and Alexa-488 staining levels were determined in these cells. Expression levels (±SD) of IFNAR1 were calculated and the results were expressed as mean fluorescence intensity (MFI) values. The p values were calculated using Student's t-test with 95% confidence intervals.

## References

[1] Drosten C, Gunther S, Preiser W, van der Werf S, Brodt HR (2003). Identification of a novel coronavirus in patients with severe acute respiratory syndrome.. N Engl J Med.

[2] Marra MA, Jones SJ, Astell CR, Holt RA, Brooks-Wilson A (2003). The Genome sequence of the SARS-associated coronavirus.. Science.

[3] Rota PA, Oberste MS, Monroe SS, Nix WA, Campagnoli R (2003). Characterization of a novel coronavirus associated with severe acute respiratory syndrome.. Science.

[4] Chan CP, Siu KL, Chin KT, Yuen KY, Zheng B (2006). Modulation of the unfolded protein response by the severe acute respiratory syndrome coronavirus spike protein.. J Virol.

[5] Tan YJ, Teng E, Shen S, Tan TH, Goh PY (2004). A novel severe acute respiratory syndrome coronavirus protein, U274, is transported to the cell surface and undergoes endocytosis.. J Virol.

[6] Law PT, Wong CH, Au TC, Chuck CP, Kong SK (2005). The 3a protein of severe acute respiratory syndrome-associated coronavirus induces apoptosis in Vero E6 cells.. J Gen Virol.

[7] Padhan K, Minakshi R, Towheed MA, Jameel S (2008). Severe acute respiratory syndrome coronavirus 3a protein activates the mitochondrial death pathway through p38 MAP kinase activation.. J Gen Virol.

[8] He B (2006). Viruses, endoplasmic reticulum stress, and interferon responses.. Cell Death Differ.

[9] Cox JS, Walter P (1996). A novel mechanism for regulating activity of a transcription factor that controls the unfolded protein response.. Cell.

[10] Hetz CA, Soto C (2006). Emerging roles of the unfolded protein response signaling in physiology and disease.. Curr Mol Med.

[11] Watowich SS, Morimoto RI, Lamb RA (1991). Flux of the paramyxovirus hemagglutinin-neuraminidase glycoprotein through the endoplasmic reticulum activates transcription of the GRP78-BiP gene.. J Virol.

[12] Xu Z, Jensen G, Yen TS (1997). Activation of hepatitis B virus S promoter by the viral large surface protein via induction of stress in the endoplasmic reticulum.. J Virol.

[13] Dimcheff DE, Askovic S, Baker AH, Johnson-Fowler C, Portis JL (2003). Endoplasmic reticulum stress is a determinant of retrovirus-induced spongiform neurodegeneration.. J Virol.

[14] Cheng G, Feng Z, He B (2005). Herpes simplex virus 1 infection activates the endoplasmic reticulum resident kinase PERK and mediates eIF-2alpha dephosphorylation by the gamma(1)34.5 protein.. J Virol.

[15] Tardif KD, Mori K, Siddiqui A (2002). Hepatitis C virus subgenomic replicons induce endoplasmic reticulum stress activating an intracellular signaling pathway.. J Virol.

[16] Pavio N, Romano PR, Graczyk TM, Feinstone SM, Taylor DR (2003). Protein synthesis and endoplasmic reticulum stress can be modulated by the hepatitis C virus envelope protein E2 through the eukaryotic initiation factor 2alpha kinase PERK.. J Virol.

[17] Benali-Furet NL, Chami M, Houel L, De Giorgi F, Vernejoul F (2005). Hepatitis C virus core triggers apoptosis in liver cells by inducing ER stress and ER calcium depletion.. Oncogene.

[18] Tardif KD, Mori K, Kaufman RJ, Siddiqui A (2004). Hepatitis C virus suppresses the IRE1-XBP1 pathway of the unfolded protein response.. J Biol Chem.

[19] Tirosh B, Iwakoshi NN, Lilley BN, Lee AH, Glimcher LH (2005). Human cytomegalovirus protein US11 provokes an unfolded protein response that may facilitate the degradation of class I major histocompatibility complex products.. J Virol.

[20] Isler JA, Skalet AH, Alwine JC (2005). Human cytomegalovirus infection activates and regulates the unfolded protein response.. J Virol.

[21] Jordan R, Wang L, Graczyk TM, Block TM, Romano PR (2002). Replication of a cytopathic strain of bovine viral diarrhea virus activates PERK and induces endoplasmic reticulum stress-mediated apoptosis of MDBK cells.. J Virol.

[22] Pestka S, Krause CD, Walter MR (2004). Interferons, interferon-like cytokines, and their receptors.. Immunol Rev.

[23] Stark GR, Kerr IM, Williams BR, Silverman RH, Schreiber RD (1998). How cells respond to interferons.. Annu Rev Biochem.

[24] Constantinescu SN, Croze E, Wang C, Murti A, Basu L (1994). Role of interferon alpha/beta receptor chain 1 in the structure and transmembrane signaling of the interferon alpha/beta receptor complex.. Proc Natl Acad Sci U S A.

[25] Basu L, Yang CH, Murti A, Garcia JV, Croze E (1998). The antiviral action of interferon is potentiated by removal of the conserved IRTAM domain of the IFNAR1 chain of the interferon alpha/beta receptor: effects on JAK-STAT activation and receptor down-regulation.. Virology.

[26] Kumar KG, Tang W, Ravindranath AK, Clark WA, Croze E (2003). SCF(HOS) ubiquitin ligase mediates the ligand-induced down-regulation of the interferon-alpha receptor.. Embo J.

[27] Kumar KG, Krolewski JJ, Fuchs SY (2004). Phosphorylation and specific ubiquitin acceptor sites are required for ubiquitination and degradation of the IFNAR1 subunit of type I interferon receptor.. J Biol Chem.

[28] Kumar KG, Barriere H, Carbone CJ, Liu J, Swaminathan G (2007). Site-specific ubiquitination exposes a linear motif to promote interferon-alpha receptor endocytosis.. J Cell Biol.

[29] Marijanovic Z, Ragimbeau J, Kumar KG, Fuchs SY, Pellegrini S (2006). TYK2 activity promotes ligand-induced IFNAR1 proteolysis.. Biochem J.

[30] Liu J, Plotnikov A, Banerjee A, Suresh Kumar KG, Ragimbeau J (2008). Ligand-independent pathway that controls stability of interferon alpha receptor.. Biochem Biophys Res Commun.

[31] Liu J, Fu WCH, Suresh Kumar KG, Qian J, Casey JP (2009). Virus-Induced Unfolded Protein Response Attenuates Antiviral Defenses via Phosphorylation-Dependent Degradation of the Type I Interferon Receptor.. Cell Host & Microbe.

[32] Law HK, Cheung CY, Ng HY, Sia SF, Chan YO (2005). Chemokine up- regulation in SARS-coronavirus-infected, monocyte-derived human dendritic cells.. Blood.

[33] Schroder M, Kaufman RJ (2005). The mammalian unfolded protein response.. Annu Rev Biochem.

[34] Xu C, Bailly-Maitre B, Reed JC (2005). Endoplasmic reticulum stress: cell life and death decisions.. J Clin Invest.

[35] Boyce M, Yuan J (2006). Cellular response to endoplasmic reticulum stress: a matter of life or death.. Cell Death Differ.

[36] Haze K, Yoshida H, Yanagi H, Yura T, Mori K (1999). Mammalian transcription factor ATF6 is synthesized as a transmembrane protein and activated by proteolysis in response to endoplasmic reticulum stress.. Mol Biol Cell.

[37] Ye J, Rawson RB, Komuro R, Chen X, Dave UP (2000). ER stress induces cleavage of membrane-bound ATF6 by the same proteases that process SREBPs.. Mol Cell.

[38] Fawcett TW, Martindale JL, Guyton KZ, Hai T, Holbrook NJ (1999). Complexes containing activating transcription factor (ATF)/cAMP-responsive-element-binding protein (CREB) interact with the CCAAT/enhancer-binding protein (C/EBP)-ATF composite site to regulate Gadd153 expression during the stress response.. Biochem J.

[39] Harding HP, Novoa I, Zhang Y, Zeng H, Wek R (2000). Regulated translation initiation controls stress-induced gene expression in mammalian cells.. Mol Cell.

[40] Ma Y, Brewer JW, Diehl JA, Hendershot LM (2002). Two distinct stress signaling pathways converge upon the CHOP promoter during the mammalian unfolded protein response.. J Mol Biol.

[41] Deshaies RJ (1999). SCF and Cullin/Ring H2-based ubiquitin ligases.. Annu Rev Cell Dev Biol.

[42] Yeh TC, Dondi E, Uze G, Pellegrini S (2000). A dual role for the kinase-like domain of the tyrosine kinase Tyk2 in interferon-alpha signaling.. Proc Natl Acad Sci U S A.

[43] Lu W, Zheng BJ, Xu K, Schwarz W, Du L (2006). Severe acute respiratory syndrome-associated coronavirus 3a protein forms an ion channel and modulates virus release.. Proc Natl Acad Sci U S A.

[44] Tan YJ (2005). The Severe Acute Respiratory Syndrome (SARS)-coronavirus 3a protein may function as a modulator of the trafficking properties of the spike protein.. Virol J.

[45] Yuan X, Yao Z, Wu J, Zhou Y, Shan Y (2007). G1 phase cell cycle arrest induced by SARS-CoV 3a protein via the cyclin D3/pRb pathway.. Am J Respir Cell Mol Biol.

[46] Bechill J, Chen Z, Brewer JW, Baker SC (2008). Coronavirus infection modulates the unfolded protein response and mediates sustained translational repression.. J Virol.

[47] Cox JS, Shamu CE, Walter P (1993). Transcriptional induction of genes encoding endoplasmic reticulum resident proteins requires a transmembrane protein kinase.. Cell.

[48] Mori K, Ma W, Gething MJ, Sambrook J (1993). A transmembrane protein with a cdc2+/CDC28-related kinase activity is required for signaling from the ER to the nucleus.. Cell.

[49] Liu CY, Wong HN, Schauerte JA, Kaufman RJ (2002). The protein kinase/endoribonuclease IRE1alpha that signals the unfolded protein response has a luminal N-terminal ligand-independent dimerization domain.. J Biol Chem.

[50] Calfon M, Zeng H, Urano F, Till JH, Hubbard SR (2002). IRE1 couples endoplasmic reticulum load to secretory capacity by processing the XBP-1 mRNA.. Nature.

[51] Lee K, Tirasophon W, Shen X, Michalak M, Prywes R (2002). IRE1-mediated unconventional mRNA splicing and S2P-mediated ATF6 cleavage merge to regulate XBP1 in signaling the unfolded protein response.. Genes Dev.

[52] Yoshida H, Matsui T, Yamamoto A, Okada T, Mori K (2001). XBP1 mRNA is induced by ATF6 and spliced by IRE1 in response to ER stress to produce a highly active transcription factor.. Cell.

[53] Chen X, Shen J, Prywes R (2002). The luminal domain of ATF6 senses endoplasmic reticulum (ER) stress and causes translocation of ATF6 from the ER to the Golgi.. J Biol Chem.

[54] Luo S, Baumeister P, Yang S, Abcouwer SF, Lee AS (2003). Induction of Grp78/BiP by translational block: activation of the Grp78 promoter by ATF4 through and upstream ATF/CRE site independent of the endoplasmic reticulum stress elements.. J Biol Chem.

[55] Wang XZ, Ron D (1996). Stress-induced phosphorylation and activation of the transcription factor CHOP (GADD153) by p38 MAP Kinase.. Science.

[56] Oyadomari S, Mori M (2004). Roles of CHOP/GADD153 in endoplasmic reticulum stress.. Cell Death Differ.

[57] McCullough KD, Martindale JL, Klotz LO, Aw TY, Holbrook NJ (2001). Gadd153 sensitizes cells to endoplasmic reticulum stress by down-regulating Bcl2 and perturbing the cellular redox state.. Mol Cell Biol.

[58] Baumeister P, Luo S, Skarnes WC, Sui G, Seto E (2005). Endoplasmic reticulum stress induction of the Grp78/BiP promoter: activating mechanisms mediated by YY1 and its interactive chromatin modifiers.. Mol Cell Biol.

[59] Hussain A, Wesley C, Khalid M, Chaudhry A, Jameel S (2008). Human immunodeficiency virus type 1 Vpu protein interacts with CD74 and modulates major histocompatibility complex class II presentation.. J Virol.

[60] Li B, Gao B, Ye L, Han X, Wang W (2007). Hepatitis B virus X protein (HBx) activates ATF6 and IRE1-XBP1 pathways of unfolded protein response.. Virus Res.

[61] Zafrullah M, Ozdener MH, Panda SK, Jameel S (1997). The ORF3 protein of hepatitis E virus is a phosphoprotein that associates with the cytoskeleton.. J Virol.

[62] Surjit M, Jameel S, Lal SK (2007). Cytoplasmic localization of the ORF2 protein of hepatitis E virus is dependent on its ability to undergo retrotranslocation from the endoplasmic reticulum.. J Virol.

[63] Barber GN (2001). Host defense, viruses and apoptosis.. Cell Death Differ.

[64] Katze MG, He Y, Gale M (2002). Viruses and interferon: a fight for supremacy.. Nat Rev Immunol.

[65] Lin RJ, Liao CL, Lin E, Lin YL (2004). Blocking of the alpha interferon-induced Jak-Stat signaling pathway by Japanese encephalitis virus infection.. J Virol.

[66] Vilcek J (2003). Novel interferons.. Nat Immunol.

[67] Ciccaglione AR, Marcantonio C, Tritarelli E, Equestre M, Vendittelli F (2007). Activation of the ER stress gene gadd153 by hepatitis C virus sensitizes cells to oxidant injury.. Virus Res.

[68] Cinatl J, Morgenstern B, Bauer G, Chandra P, Rabenau H (2003). Treatment of SARS with human interferons.. Lancet.

[69] Padhan K, Tanwar C, Hussain A, Hui PY, Lee MY (2007). Severe acute respiratory syndrome coronavirus Orf3a protein interacts with caveolin.. J Gen Virol.

[70] Shen J, Prywes R (2005). ER stress signaling by regulated proteolysis of ATF6.. Methods.

[71] Lai WL, Wong NS (2005). ROS mediates 4HPR-induced posttranscriptional expression of the Gadd153 gene.. Free Radic Biol Med.

[72] Yoshida H, Haze K, Yanagi H, Yura T, Mori K (1998). Identification of the cis-acting endoplasmic reticulum stress response element responsible for transcriptional induction of mammalian glucose-regulated proteins. Involvement of basic leucine zipper transcription factors.. J Biol Chem.

[73] Chaudhary A, Das SR, Hussain A, Mayor S, George A (2005). The Nef protein of HIV-1 induces loss of cell surface costimulatory molecules CD80 and CD86 in APCs.. J Immunol.

[74] Hussain A, Das SR, Tanwar C, Jameel S (2007). Oligomerization of the human immunodeficiency virus type 1 (HIV-1) Vpu protein–a genetic, biochemical and biophysical analysis.. Virol J.

[75] Kar-Roy A, Korkaya H, Oberoi R, Lal SK, Jameel S (2004). The hepatitis E virus open reading frame 3 protein activates ERK through binding and inhibition of the MAPK phosphatase.. J Biol Chem.

[76] Mukherji A, Janbandhu VC, Kumar V (2007). HBx-dependent cell cycle deregulation involves interaction with cyclin E/A-cdk2 complex and destabilization of p27Kip1.. Biochem J.

[77] Lin JH, Li H, Yasumura D, Cohen HR, Zhang C (2007). IRE1 signaling affects cell fate during the unfolded protein response.. Science.

